# Type 2 diabetes mellitus-related environmental factors and the gut microbiota: emerging evidence and challenges

**DOI:** 10.6061/clinics/2020/e1277

**Published:** 2020-01-06

**Authors:** Yanfen Liu, Xueyong Lou

**Affiliations:** Jinhua Municipal Central Hospital, Department of Endocrinology Jinhua, 321000, China

**Keywords:** Type 2 Diabetes Mellitus, Gut Microbiota, Environmental Factors

## Abstract

The gut microbiota is a group of over 38 trillion bacterial cells in the human microbiota that plays an important role in the regulation of human metabolism through its symbiotic relationship with the host. Changes in the gut microbial ecosystem are associated with increased susceptibility to metabolic disease in humans. However, the composition of the gut microbiota in those with type 2 diabetes mellitus and in the pathogenesis of metabolic diseases is not well understood. This article reviews the relationship between environmental factors and the gut microbiota in individuals with type 2 diabetes mellitus. Finally, we discuss the goal of treating type 2 diabetes mellitus by modifying the gut microbiota and the challenges that remain in this area.

## INTRODUCTION

Diabetes mellitus (DM) is one of the most prevalent diseases worldwide. Type 2 diabetes mellitus (T2DM) is a syndrome induced by insufficient insulin secretion or impaired insulin secretion, which constitutes the majority of cases; T2DM has become a serious threat to public health and is a growing burden on global economies (1). This disease seriously affects people’s quality of life due to several severe complications (2). It has been estimated that the number of T2DM patients will increase from 450 million in 2016 to 642 million in 2040 (3). In addition to genetic factors, T2DM is also affected by environmental factors, which suggests that diet and obesity, among other factors, are involved in increasing the risk of diabetes (3-5). These environmental risk factors can also lead to diabetes by changing the gut bacterial microbiota (6,7).

The bacterial microbiota is the best studied component of the gut microbiota, which inhabits the host at different concentrations. The gut bacterial microbiota shows differences in the concentration gradient from the mucosa to the lumen and proximally to distally, showing significant differences between individuals (8-10). The gut bacterial microbiota evolves from a poorly differentiated community at birth into a highly complex community (11,12). Accumulating evidence supports a view of the gut microbiota in the development of metabolic diseases, including type 2 diabetes (13-18), illustrating the differences between the gut microbiota in T2DM patients and healthy individuals (18,19). The new evidence suggests that the adaptive capacity of the gut microbiome can be used to develop better programmes for the prevention and treatment of T2DM (20). The relationship between the gut microbiota and DM has not been systematically reviewed in the literature, in which data are sometimes contradictory or one-sided. Therefore, whether changing environmental factors such as diet and exercise in diabetic patients can change the gut microbiota to prevent and treat diabetes is a matter of debate. This review summarizes the relationship between the gut microbiota and T2DM-related environmental factors.

## THE GUT MICROBIOTA

The gut microbiota refers to all the parasitic microflora in the intestine, which include a variety of bacteria, fungi, and protozoa. The majority of the gut microbiota consists of five phyla, namely, Bacteroidetes, Firmicutes, Actinobacteria, Proteobacteria, and Verrucomicrobia (21), which play a pivotal role in protecting the host against pathogenic microbes (22,23); the gut microbiota has a profound influence on modulating immunity (24,25) and regulating metabolic processes (26,27).

There are approximately 38 trillion bacterial cells in the human microbiome, and there are 30 trillion human cells in the body (28). Three main phyla are colonized in the intestine, namely, Phytoplasma, Bacteroidetes and a small number of actinomycetes. In humans, studies have shown that an increase in sclerenchyma and a decrease in *Bacteroides* are positively associated with obesity (29,30). Although its importance has not been fully recognized until recently, the intestinal tract has a multilevel ability to influence glucose homeostasis, which is essential for nutrient absorption and transport to different organs and tissues of the human body (31). The human gastrointestinal tract constitutes a part of the body with a particularly high density of immune cells, and microorganisms colonize the intestine at birth. Influenced by a variety of environmental factors, the role of microorganisms in the process of immune system initiation has attracted extensive attention (32,33). More than 70% of microbes live in the gastrointestinal tract and establish reciprocal relationships with their hosts, from the gastric cavity to the small intestine to the rectum, where they reach maximum concentrations. The microbiota is considered a metabolically active organ (34). Mainly, the symbiotic intestinal bacteria Bacteroidetes, Firmicutes and Actinomycetes obtain energy from the fermentation and transformation of undigested food substrates (35). The microbiota can be regarded as an organ in which “the new supersedes the old”; the microbiota can accurately meet our physiological needs, and we do not need to evolve to receive dietary energy from lean-associated microbiomes.

There are more than 1,000 kinds of microorganisms in the human intestinal tract, representing hundreds of species and thousands of subspecies. The average human intestinal microbiome comprises approximately 160 bacterial species in each individual (21). The microbial community mainly resides in the gastrointestinal tract, especially in the colon, which is mainly anaerobic and has a rich nutritional environment that is the preferred site for intestinal microbial colonization. The microorganisms interact with the host and each other to affect the host’s physiology and health. The modification of the intestinal microflora as a potential treatment for human and animal diseases has attracted increasing attention.

## DIABETES-RELATED ENVIRONMENTAL FACTORS AND THE GUT MICROBIOTA

### Diet, the Gut Microbiota and DM

Diet is one aspect of the environment that directly affects the gut microbiota (36); this effect occurs because changes in microbial composition can cause insulin resistance, inflammation, and vascular and metabolic disorders. Diets rich in carbohydrates and simple sugars lead to the increased proliferation of Firmicutes and Proteobacteria, while diets rich in saturated fat and animal protein favour the proliferation of Bacteroidetes and Actinobacteria (37). Some ground-breaking concepts suggest that non-pathogenic gut bacteria are more beneficial to human health than other bacteria, and in 1908, when Elie Metchnikoff proposed that the microbiome could extend life and stave off old age and decline, he recommended the regular consumption of milk artificially acidified with *Lactobacillus delbrueckii* subsp. *bulgaricus*, the source of the probiotic craze (38,39).

New evidence suggests that altering the intestinal flora is important for the prevention and treatment of T2DM (7). A rapid increase in the prevalence of T2DM worldwide is related to rapid changes in the environment, which have a negative impact on the risk factors for diabetes; these environmental changes include changes in dietary habits in particular, which modulate gut microbiome composition largely by regulating excessive biological functions (40-42). It has been found that the best choices of dietary factors play a critical role in preventing early T2DM and reducing the risk of lifetime T2DM (43). The gut environment is affected by diet, including the absorption of micronutrients and nutrients, and changes in pH, which in turn change the balance of the gut microbiota (44). Intestinal pH plays an important role in the composition of resident bacteria. For example, at pH 5.5, butyrate-producing *Phytophthora* accounts for 20% of the total bacteria, while at pH 6.5, the number of butyrate-producing *Phytophthora* decreases, while the number of acetate- and propionate-producing *Phytophthora* increases (45).

Several studies have shown that patients with DM demonstrate increased permeability of butyrate secreted by intestinal epithelial cells, and butyrate is the main source of energy for intestinal epithelial cells (46-50); therefore, impaired butyrate secretion is one of the reasons for the loss of the tight barrier function of intestinal epithelial cells (51). The intestinal microflora can be used to understand individual responses to dietary interventions (52).

Epidemiological studies have consistently shown a negative correlation between dietary fibre consumption and the incidence of T2DM. Dietary fibre and whole grains have been shown to increase the diversity of the intestinal microflora in humans (53,54). High fibre intake has been shown to be associated with increased levels of *Prevotella* bacteria in several studies (41,42,55), and a high-fat diet (HFD) has been shown to alter the metabolic activity of the mammalian gut microbiome (41).

Studies have shown that a HFD can lead to changes in major intestinal flora, such as *Bifidobacterium* and *Bacteroides*, resulting in an increase in the proportion of Gram-negative bacteria/Gram-positive bacteria. This significant change is associated with increased plasma lipopolysaccharide, fat content and body weight; the accumulation of triglycerides in the liver; DM; and inflammatory reactions (56). The clinical effect of gegen decoction in different doses on T2DM was evaluated. With the increase in the dose of the drugs, a significant increase in the number of the eubacterium *Hodgsonia*, which is closely related to improvement of DM, was found in the faecal microbiome. The greater the level of the bacteria, the better the blood sugar control (57).

The microbiome, which is stable and resilient to environmental disturbances (such as changes in the diet or short-term antibiotic exposure) (58), also plays roles in the epigenetic regulation of host genes. Recent studies have shown that the intestinal microflora, as an epigenetic regulator, affect host metabolism by modifying DNA methylation (59). Therefore, the effective regulation of the intestinal microflora may be a promising strategy for the treatment of metabolic disorders, including DM. One study demonstrated that NLRP12/ mice fed a HFD as well as sumac were deficient in *Clostridium difficile*. The low number of these bacteria has been proposed as a marker for increased inflammatory bowel disease in children (60). The influences of diet on the composition of the microbiota were found to control body weight after bariatric surgery, regulate plasma glucose and insulin levels, maintain intestinal epithelial barrier integrity, and reduce the levels of inflammatory cytokines (61-64).

**Figure 1:**
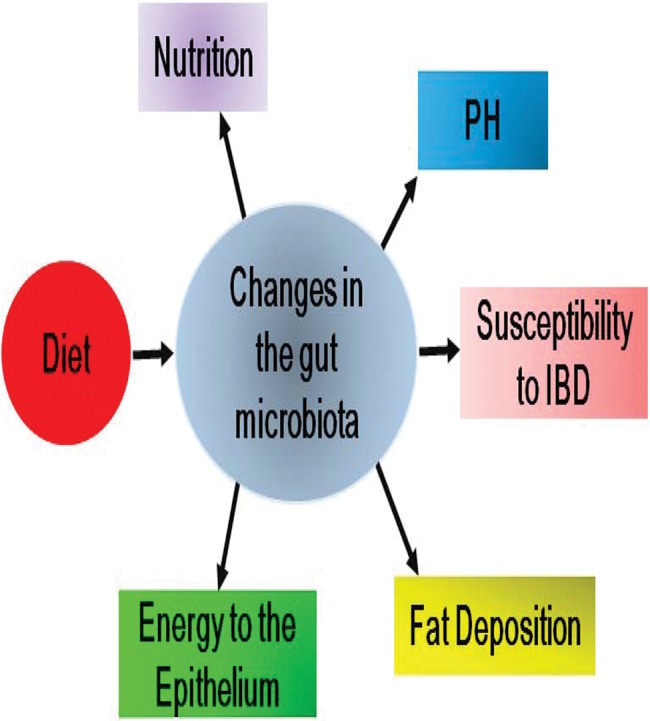
Effects of diet on the composition of the gut microbiota and subsequent pathophysiological changes during DM progression.

The effects of diet on the composition of the gut microbiota and the subsequent pathophysiological changes during DM progression are illustrated in Figure 1.

### Obesity, the Gut Microbiota and DM

The gut microbiota plays an important role in obesity, one of the main risk factors for metabolic syndrome. The current global obesity epidemic is increasing, leading to an increase in the incidence and prevalence of T2DM by reducing insulin sensitivity in the adipose tissue, liver and skeletal muscle and subsequently impairing beta-cell function, which poses a serious challenge to the healthcare system (65,66). Unexpectedly, the gut microbiota is strongly correlated with host metabolism and weight gain and can be a positive driver of obesity, in which an imbalance is associated with intestinal inflammation (67-69). In a C57BL/6 mouse study, in which even when fed a high-fat/high-carbohydrate diet the mice did not develop obesity due to genes that protect mice from obesity, the obese mouse microbiota that accumulated in these mice led to a significant increase in body fat; these mice, despite the reduced food intake, still demonstrated insulin resistance (70-72). In a double-blind randomized controlled intervention study, probiotics, such as *Lactobacillus bulgaricus*, were found to significantly reduce body weight in overweight and obese subjects (73). In a study of 18 lean and 18 overweight males with T2DM, while the bacterial abundance was similar in both groups, the abundance of Firmicutes bacteria was significantly higher in controls than in participants with T2DM (74).

Disorders of the gut microbiota are associated with T2DM, insulin resistance and obesity (75-78). Obesity increases intestinal permeability and the possibility of organic acids such as succinic acid being released by intestinal symbiotic bacteria into circulation (79). Obesity is associated with changes in the relative abundance of the two dominant bacterial divisions, Bacteroidetes and Firmicutes (80). A sterile adult mouse distal intestinal microbial community was obtained from conventionally bred mice, and the colonization significantly increased body fat in 10-14 days, although the relative food consumption was reduced (72). This change was related to several interrelated mechanisms (the microbial fermentation of dietary polysaccharides can be performed by the host in the liver, where they are transformed into more complex lipids and the regulation of microbes by host genes); these findings lead us to propose that in obese individuals, specific microorganisms extract energy from the diet more effectively than a group of microorganisms in lean individuals (81). Individuals’ gut microbes vary in abundance and proportion due to differences in long-term eating habits (42).

Using a mouse model, Gordon’s team was the first to identify ways in which the gut microbiota can affect host metabolism, which may affect obesity (70,72). The comparison between obese mice and lean mice showed that the number of Gram-negative bacilli in obese mice decreased by 50%, while the number of Gram-positive bacilli increased (70). The higher the *Bacteroides* level, the lower the body weight (30,82). Weight loss in overweight and obese adolescents led to an increase in *Bacteroides* (83), which reduced the incidence of diabetes (84). Bariatric surgery leads to long-term increases in the protein and bile acids involved in improving glucose metabolism (7). The mechanism is unknown, but it is likely due to physiological changes after surgery. The “lean” phenotype was transferred by transplanting the patient’s gut microbiota into germ-free (85). There was a negative correlation between the level of *Faecalibacterium prausnitzii* and inflammatory markers, suggesting that* Faecalibacterium prausnitzii* may regulate systemic inflammation in obese diabetic patients and contribute to the improvement in diabetes mellitus (27).

### Relationship between Glucose-Lowering Drugs and the Gut Microbiota in Type 2 Diabetes Mellitus

Hypoglycaemic agents can affect the composition of the intestinal microflora, and acarbose increases the relative abundance of lactic acid bacteria and bifidobacteria in the intestinal flora and consumes *Bacteroides*, thus altering the relative abundance of bacteria related to bile acid metabolism. Faecal samples were transferred from metformin-treated donors to sterile mice before and after treatment for 4 months. Glucose tolerance was improved in mice receiving metformin-modified microbiota (86,87). In addition, changes in the gut microbiota can reduce the adverse reactions to hypoglycaemic drugs (88).

Metformin mainly accumulates in the intestine, and its concentration in the intestine is approximately 300 times higher than that in plasma. Therefore, the intestinal tract is the main location of its hypoglycaemic action (89,90). Currently, it is still very difficult to formulate strategies to regulate the composition of microflora and guide its metabolic effect from the perspective of diabetes prevention or treatment. Nevertheless, a growing body of literature offers some insights into the potential use of the microbiota as a therapeutic target for diabetes. *Lactobacillus* and *Bifidobacterium* are the most studied and used probiotics. Probiotics are defined as “dietary fibre with recognized positive effects on intestinal flora” (91-93). They are also used to prevent intestinal flora disorders caused by antibiotic treatment after infection with *Clostridium difficile* (94). The effect of *Lactobacillus rhamnosus* on glucose and glucose tolerance in streptozotocin (STZ)-induced diabetic rats was studied. Glucose intolerance and hyperglycaemia may be delayed in sick rats treated with *Lactobacillus* strain GG. Therefore, bifidobacteria can reduce intestinal fat polysaccharide levels and improve the function of the intestinal barrier (95).

A study was conducted with 20 volunteers with T2DM and a daily intake of 200 mL of synbiotics containing *Lactobacillus acidophilus*, *Bifidobacterium* and fructooligosaccharides. However, after consuming the synbiotic shake for one month, the volunteers who had ingested the shake had significantly higher levels of high-density lipoprotein cholesterol (HDL-c) and significantly lower blood sugar levels (96). Compared with the microbiota of individuals with non-metformin-treated DM, the microbiota of individuals with metformin-treated DM was significantly different only at the bacterial family level (PERMANOVA FDR <0.1), indicating the effect of metformin treatment on intestinal microorganisms. Univariate testing of the efficacy of metformin showed a significant increase in *Escherichia* spp. and a reduced abundance of *Intestinibacter* (20). Microbes that reside in the human gut are key contributors to host metabolism and are considered potential sources of novel therapeutics. The colonization of intestinal microorganisms in the first few years of life is obviously crucial to the development of host immune regulation. A disturbance in microbial community composition or host response may lead to chronic inflammation (97). Intestinal microbial ecosystems may be out of balance due to the overgrowth of some microorganisms, which is defined as intestinal dysbiosis.

Forslund and his colleagues found that there were fewer butyrate-producing bacteria in T2DM patients who did not receive metformin treatment than in the control individuals who did not have diabetes. They also clarified that the increase in lactic acid bacteria in previously diagnosed T2DM patients was the result of metformin treatment (20). The pharmacological effects of metformin include bile acid recycling and changes in the gut microbiota, which promote the secretion of glucagon-like polypeptide-1 (GLP-1) (98). It was found that metformin had a significant effect on the intestinal flora composition. Patients with T2DM who took metformin could be identified by changes in intestinal flora composition (20).

Several treatments are used to treat insulin resistance and diabetes, but metformin is the most popular first-line drug, and some of its beneficial effects can be attributed to changes in the gut microbiota. In addition to improving blood sugar levels in mice fed a HFD, metformin treatment also increased the number of mucoprotein-degrading bacteria called *Akkermansia* bacteria. Population studies in Denmark, Sweden and China confirmed this finding (20,99-101). The gut microbiota regulates the intestinal barrier and inflammation through metabolites such as short-chain fatty acids (SCFAs) (36). Compared with non-diabetic patients, diabetic patients taking metformin had increased mucosal eosinophils and bacteria producing SCFAs (99). Experimental studies have shown that the abundance of mucus *Akkermansia* and *cocleatum* in HFD-fed mice treated with metformin increased significantly (101,102). The abundance of *Intestinibacter* spp. in diabetic patients treated with metformin decreased, while the *Escherichia coli* abundance increased (20). *Adlercreutzia* spp. in the faeces of diabetic patients treated with metformin alone increased (98). These results indicate the effect of metformin on intestinal microorganisms in patients.

As a classic alpha-glucosidase inhibitor, acarbose can inhibit the enzymes that breakdown oligosaccharides into monosaccharides and disaccharides in the intestinal tract, thus delaying the absorption of postprandial glucose (103). Ninety-five T2DM patients were divided into two groups: one group was treated with acarbose while the other was not treated with acarbose; after treatment with acarbose, the *Bifidobacterium* content was lower and that of *Enterococcus faecalis* was higher (104). These results suggest that acarbose treatment could alter the gut microbiota. Acarbose regulates bile acid metabolism by increasing the relative abundance of lactic acid bacteria and bifidobacteria in the intestinal flora, which has beneficial effects on host metabolism (86). Glp-1 is known as an intestinal hormone involved in glucose metabolism, appetite regulation and gastric emptying. The ability of the gut microbiota to accelerate gastrointestinal motility is mainly due to the inhibition of gastrointestinal glp-1 receptor expression (105). Glp-1 sensitivity is regulated by intestinal-flora-dependent regulation in the intestinal nervous system (106). The abundance of *Akkermansia muciniphila* was found to be decreased in individuals with obesity and diabetes mellitus (13). Sitagliptin is a dipeptidyl-4 (dpp-4) inhibitor that has also been found to improve intestinal microbial structure, which can be mediated by reducing intestinal inflammation and maintaining intestinal mucosal barrier integrity (62). Vildagliptin can significantly reduce the diversity of the microbiota of diabetic rats and normalize the Bacteroides-Prevotella ratio (107). These studies are of great significance for understanding the role of the intestinal microflora as a new target for diabetes treatment. However, further research is needed to confirm this hypothesis.

The effects of these drugs on the composition of the gut microbiota are summarized in Table 1.

## THE GUT MICROBIOME: CONCLUSIONS AND FUTURE PERSPECTIVE

We are living with a tremendous number of microorganisms in our guts. In recent years, an increasing number of studies have shown that the intestinal microflora is directly related to the occurrence and development of diabetes. The related topic of the gut microbiota has become a research hotspot, and the relationship between DM and the intestinal flora has the potential to promote clinical trials and drug development and improve the effectiveness of treatment strategies. An imbalance in the gut microbiota composition can lead to several diseases. However, there are still some problems to be further studied, such as the specific molecular mechanism by which the gut microbiota affects DM and the treatment of T2DM by altering the gut microbiota.

Regarding the aetiology of DM, many factors have been clarified in medicine. With the in-depth study of the intestinal flora, people have a new understanding and expansion of the aetiology, which also provides public guidance for the better prevention of obesity and diabetes. The intestinal flora plays an important role in maintaining human health. Metabolic disorders can lead to intestinal flora imbalance, which will further aggravate metabolic disorders, thus forming a vicious cycle. Current methods of adjusting intestinal flora homeostasis include modifying dietary components using probiotics or probiotics and faecal transplantation. The relationship between environmental factors involved in DM and the gut microbiota is not fully understood. Understanding the influence of environmental factors on intestinal microflora is of great significance for increasing the understanding of the aetiology, diagnosis, treatment, adverse reactions and prognosis of metabolic diseases.

With the steady increase in the prevalence of T2DM, new treatments are needed that can not only temporarily improve blood glucose control but also change the course of diabetes. Increasing evidence suggests that the gut microbiota plays an important role in the pathogenesis of DM. The relationship between hypoglycaemic agents and the gut microbiota is not fully understood. The key regulatory role of the gut microbiota and its contribution to T2DM were further emphasized through the changes in intestinal microbial ecology observed after environmental factors such as diet, fat and drug interventions were altered; however, the mechanism that underlies the effects on the composition of the intestinal flora is still unclear. In the future, it is necessary to study the relationship between the intestinal flora and T2DM from the perspective of molecular biology and to develop drugs based on the direct and precise genetic modification of the microbiome to provide guidance for the better prevention and treatment of T2DM. Probiotics regulate the imbalance of the intestinal microbiome composition by increasing the bacterial population, intestinal epithelial barrier function, and cytokine production.

## AUTHOR CONTRIBUTIONS

Liu Y participated in the conceptual design and writing of the paper. Lou X was responsible for the critical revision of the manuscript for important intellectual content.

## Figures and Tables

**Table 1:** Effects of glucose-lowering drugs on the gut microbiota.

Drug	Change in the microbiota	Effects on DM	Possible mechanisms
Acarbose	lactic acid bacteria↑ Bifidobacteria↑ Bacteroides↓	Protective against DM	Alters bile acid metabolism
Probiotics	Bifidobacteria↑	Enhanced epithelial permeability	Reduces levels of polysaccharide
Metformin	lactic acid bacteria↑	Protective against DM	Alters bile acid metabolism
Metformin	Akkermansia bacteria↑	Enhanced epithelial permeability	Increases levels of SCFAs
Acarbose	Bifidobacterium↓ Enterococcus↑ lactic acid bacteria↑	Protective against DM	Alters bile acid metabolism
